# An approach to comparing tiling array and high throughput sequencing technologies for genomic transcript mapping

**DOI:** 10.1186/1756-0500-2-150

**Published:** 2009-07-24

**Authors:** Rajkumar Sasidharan, Ashish Agarwal, Joel Rozowsky, Mark Gerstein

**Affiliations:** 1 Molecular Biophysics and Biochemistry Department, Yale University, New Haven, CT 06520, USA; 2 Department of Plant Biology, Carnegie Institution for Science, Stanford, California 94305, USA; 3 Interdepartmental Program in Computational Biology and Bioinformatics, Yale University, New Haven, CT 06520, USA; 4 Department of Computer Science, Yale University, New Haven, CT 06520, USA

## Abstract

**Background:**

There are two main technologies for transcriptome profiling, namely, tiling microarrays and high-throughput sequencing. Recently there has been a tremendous amount of excitement about the latter because of the advent of next-generation sequencing technologies and its promises. Consequently, the question of the moment is how these two technologies compare. Here we attempt to develop an approach to do a fair comparison of transcripts identified from tiling microarray and MPSS sequencing data.

**Findings:**

This comparison is a challenging task because the sequencing data is discrete while the tiling array data is continuous. We use the published rice and *Arabidopsis *datasets which provide currently best matched sets of arrays and sequencing experiments using a slightly earlier generation of sequencing, the MPSS tag sequencing technology. After scoring the arrays consistently in both the organisms, a first pass comparison reveals a surprisingly small overlap in transcripts of 22% and 66% respectively, in rice and *Arabidopsis*. However, when we do the analysis in detail, we find that this is an underestimate. In particular, when we map the probe intensities onto the sequencing tags and then look at their intensity distribution, we see that they are very similar to exons. Furthermore, restricting our comparison to only protein-coding gene loci revealed a very good overlap between the two technologies.

**Conclusion:**

Our approach to compare genome tiling microarray and MPSS sequencing data suggests that there is actually a reasonable overlap in transcripts identified by the two technologies. This overlap is distorted by the scoring and thresholding in the tiling array scoring procedure.

## Background

Although gene expression analysis can reveal interesting clues to the biochemical state of cells, they do not provide a complete picture of cellular transcription as they focus only on known protein-coding genes. There has been considerable interest in the identification and implication of new non-coding RNA molecules in myriad cellular functions [1]. Therefore, it is essential that we use unbiased technologies for transcript mapping to expand our understanding of these classes of RNAs. The introduction of ultra high-throughput next-generation DNA sequencing technologies in the sequencing market at this juncture offers a unique opportunity to develop methods for a fair comparison of data from these technologies.

In this work, our objective is to describe an approach towards comparing tiling microarray data and sequencing data for genome-wide transcript mapping. Although the ideal comparison would be using data from a high-resolution tiling array and next-generation sequencing methods, the best available datasets at this point of time are the 36-mer oligo nucleotide arrays (positioned every 46 nucleotides) and the tag based MPSS (Massively Parallel Signature Sequencing) technology. We explore how well these two technologies compare in transcript detection for *Arabidopsis *and rice, the two most well-studied model organisms for understanding plant biology.

This objective is not a trivial task as we must bear in mind that the nature of the two types of data that we are comparing is completely different. The data from sequencing experiments are simple, in that, they are discrete and provide a start and an end coordinate for transcripts. Tiling array experiments provide a continuous value, the intensity measure, for each probe on the array. These probes correspond to a discrete sampled genomic region and transcript boundaries from a collection of probe intensities are then identified by applying segmentation methods on these data points. Typically, the methods employed for demarcating transcript boundaries have to deal with issues of choosing correct thresholds. One of the algorithmic challenges in analyzing tiling array data is choosing an optimal set of parameters that would reduce the number of false positives when scoring transcriptionally active regions. We rescored the Arabidopsis and rice tiling array datasets using a uniform set of parameters (Additional file 1).

### Overlap in transcripts identified using MPSS and tiling microarray data

**Figure 1:**
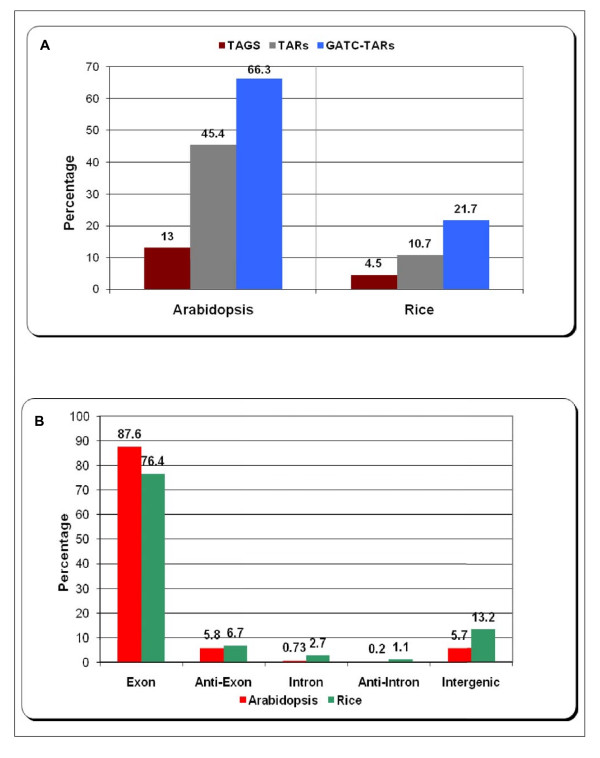
**Intersection of MPSS tags and TARs from tiling microarray for *Arabidopsis *and rice**. Figure 1A shows the percentage of transcripts that are identified by both the technologies. GATC-TARs represent the percentage over the subset of TARs that contain at least one GATC motif, the recognition site for the anchoring enzyme DpnII used in MPSS sequencing protocol. Figure 1B shows the distribution in percentage of the transcripts that are identified by both the technologies among five classes of genomic features, *viz*. exon, anti-exon, intron, anti-intron and intergenic.

After identifying transcripts from tiling array data we compared the extent to which the two platforms overlap in their transcriptome profiling. A simple intersection of MPSS tags and tiling array TARs (Transcriptionally Active Regions) shows that they overlap poorly. 13% of MPSS tags (16,647) overlapped with 45% of TARs (11,207) in *Arabidopsis *while only 4.5% (4,513) of MPSS tags overlapped with 10.7% of TARs (3,554) in rice (Figure 1A). An inherent feature of MPSS technology is its inability to identify transcripts that do not have a restriction site recognized by the anchoring enzyme DpnII (recognition site: GATC). We observed that the overlap increases to 66.3% of all TARs for *Arabidopsis *when we consider only those TARs that contain at least one occurrence of the tetra-nucleotide GATC. Ideally, only these TARs will have a chance of overlapping with the MPSS tags. There were 16,902 TARs out of a total number of 24,712 TARs in *Arabidopsis *that had at least one GATC. There are 16,392 TARs in rice that have at least one GATC and if we consider only these, the overlap increases from 10.7% to 21.7%.

As we expected, a large percentage of the overlap is in exonic transcripts – 87.6% and 76.4% in *Arabidopsis *and rice, respectively (Figure 1B). The percentage of overlap is ~6% in *Arabidopsis *and ~7% in rice for anti-sense exonic transcripts and ~6% in *Arabidopsis *and ~13% in rice for intergenic transcripts. Thus, in terms of confirming TARs using a second method, there is a moderate overlap between MPSS tags and *Arabidopsis *TARs (66.3%) but a low overlap for rice TARs (21.7%). In this process, we found ~1000 novel transcripts in *Arabidopsis *and ~600 novel transcripts in rice that were identified by both the platforms. These represent a highly concordant set of intergenic transcripts for these two species. A comparison of protein-coding gene loci identified by the two technologies revealed a very good overlap (Additional file 2).

### Calculating mean intensity for MPSS tags from tiling microarray data

We sought to investigate the reason behind the low to moderate overlap for MPSS tags. This objective also addresses a related question, namely, identifying the right set of parameters that would maximize the overlap. The three key parameters, namely, signal intensity threshold, *maxgap *and *minrun *can be tweaked to generate multiple sets of TARs. We reasoned that we are actually comparing two completely different types of data – the end product of a sequencing run is a 17 base pairs (bp) tag that has start and end coordinates in genomic space. The TARs have a start and an end coordinate too, but are derived from processing a set of continuous values in the form of intensity measure for the probes spotted on the array. The transcript boundaries, in effect, reflect the probe geometry rather than the actual start and end of a transcript. Thus, to accomplish a fair comparison of data from the two methods we decided to look at the distribution of probe intensities around the MPSS tag location after mapping the tags on the tiling array.

In order to map MPSS transcripts with probe intensities on the tile path, we followed a simple procedure to assign normalized intensities for tags. First, we converted raw intensities from the array into intensity percentiles. Intensity percentiles let us compare the intensity scales across multiple slides in a uniform way. For each tag, we calculated the mean of intensity percentiles from probes that lie within its start and end co-ordinates. Since MPSS tags are only 17 nt in length, we considered flanks of increasing nucleotide lengths, *viz*, 25, 50, 75 and 100. Along with the MPSS tag, these flanks translate to, on average, 2, 3, 5 and 6 probes, respectively. These regions correspond to, 67, 117, 167 and 217 bp in length, respectively, on the tile path. In order to evaluate the intensity distribution that we obtained from empirical data with random expectation, we chose 10,000 regions randomly from the tile path and calculated the mean intensity in percentile for these regions.

### MPSS tags are enriched for higher intensities on the tiling microarray

**Figure 2:**
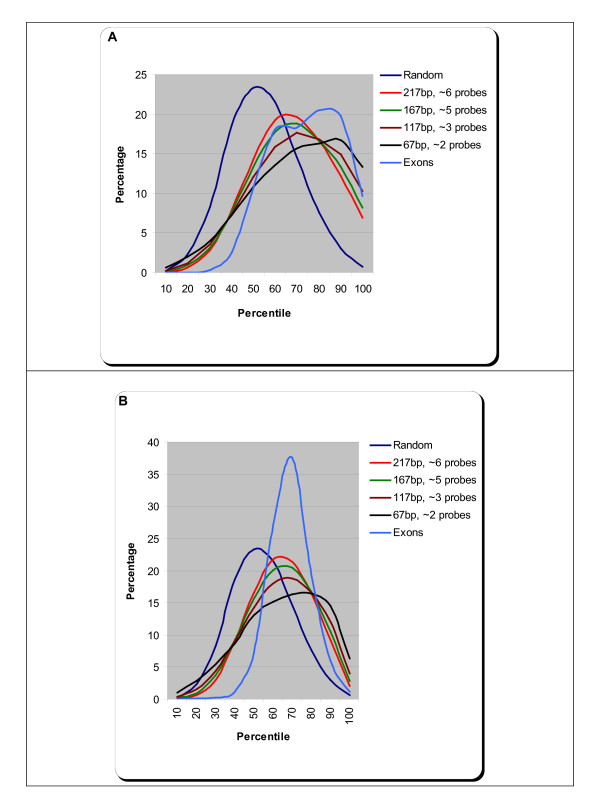
**Distribution of intensities for all MPSS tags in *Arabidopsis *and rice**. The 128,337 reliable MPSS tags in *Arabidopsis *were mapped to the probes on the tiling microarray. For each tag, we calculated the mean intensity, after converting the raw intensities into percentiles, from probes that lie within the start and end coordinates of an MPSS tag. Since MPSS tags are only 17 nt in length, we considered flanks of increasing nucleotide lengths, *viz*, 25, 50, 75 and 100. Along with the MPSS tag, these flanks translate to, on average, 2, 3, 5 and 6 probes, respectively. These regions correspond to, 67, 117, 167 and 217 bp in length, respectively, on the tile path. The plot in Figure 2A shows the percentage of MPSS tags for different bins of percentiles. A similar procedure was followed for calculating intensities for the 100,274 reliable MPSS tags in rice. The distribution of intensities for rice is shown in Figure 2B.

Figure 2 summarizes the results we obtained from implementing the above procedure. Figure 2A shows the distribution of intensities for, the four different tag flanks, all probes within exons and for the 10,000 randomly selected regions on the tiling array for *Arabidopsis*. Figure 2B shows the corresponding set of distribution of intensities for rice. An unpaired *t-test *on the intensity distributions of MPSS tags for each of the four regions and the random distribution gave a *p-value *less than 2.2e–16 suggesting that intensity distributions from probes that map to the same region as the tags are significantly different from the random distribution; in addition, the distributions overlap with intensity distribution from exonic probes. We also observed that there is a significant enrichment in signal on the tiling array in the immediate vicinity of the MPSS tag.

In *Arabidopsis*, the mean intensity percentile of the region around the tag increases from 63.1 when we consider a region spanning 6 probes on average to 66.1 when we consider only the immediate probe or two. The mean intensity percentile of all exonic probes is 70.8 in *Arabidopsis*. Thus, if we consider a signal intensity percentile of 70 as our threshold intensity for calling transcription in *Arabidopsis*, we find that 62% of all MPSS tags show an enriched signal similar to transcribed exons.

In rice, the mean intensity percentile increases from 59.9 to 61.1 as we go from 6 probes to 2 probes around the MPSS tag. An unpaired *t*-test gave a *p-value *less than 2.2e–16 suggesting that the mean intensity percentile obtained from 2 probes is significantly higher than the mean intensity obtained from 6 probes. This holds good for both *Arabidopsis *and rice. The mean intensity percentile of all exonic probes in rice is 64.6. Again, if we consider a signal intensity of 65 as threshold intensity for transcription in rice, we find that 61.2% of MPSS tags lie above this threshold. In both the organisms, the mean intensity percentile of a collection of probes from regions that are chosen randomly would be 50. Thus, although a simple intersection of transcripts obtained from the MPSS platform and genome tiling microarray did not show a great overlap, we were able to demonstrate that ~62% of these MPSS tags in both *Arabidopsis *and rice are in fact enriched for higher intensity on the tiling array. This observation holds good for both the unique MPSS tags (Additional file 3) and for all tags as described above.

### MPSS tag abundance measure and tiling array intensity are not correlated

We used the mean intensity percentile for each of the MPSS tags described above to see how well this measure of hybridization correlates with the abundance measurement of the respective tags obtained from the MPSS experiments. There are 17 libraries in *Arabidopsis *and 22 libraries in rice with an abundance measurement for all the reliable MPSS tags. In order to be conservative we considered only tags that map to a unique genomic location. We performed regression of the log_2 _transformed abundance values against the intensity percentile for unique MPSS tags in both rice (Additional File 4) and *Arabidopsis *(Additional File 5) and observed that the two measures of transcript levels are not correlated. This observation was consistent when we calculated the regression for individual libraries, pooled libraries and also for MPSS tags that overlap with tiling array TARs from the two species. The correlation coefficient for the pooled 17 libraries in *Arabidopsis *was 0.27 (range, 0.15–0.42) and 0.13 for MPSS tags that overlap with TARs. For rice, the correlation coefficient for the pooled 22 libraries was 0.25 (range, 0.13–0.26) and 0.13 for tags that overlap TARs.

## Conclusion

Our approach presents a novel way of looking at these two different types of data. While an overlap from a naïve comparison of identified transcripts between these two technologies is not as high as one would have expected, it is not altogether surprising. The transcriptionally active regions identified on the tiling array are obtained after processing the intensities from the probes. During this process, we actually convert continuous-value probe intensity values into discrete genomic regions (TARs). The sequencing data is already discrete, represented by the 17 bp tag that maps to a genomic location. Facilitating such a comparison by mapping tags to probe intensities directly, however, does improve the correlation between the two technologies significantly. We believe our approach is general purpose and should work on next generation sequencing data.

The TAR identification procedure that we followed implies that unless a region has a set of consecutive high intensity probes it is not likely to be considered as a feature. Also, shorter transcripts are likely to be missed. The *maxgap *parameter in our procedure demarcates transcript boundaries when there is a gap of more than 20 nucleotides between consecutive probes. This is likely to be an important issue in analyzing the rice tiling array data for the following reasons. The rice tiling array was designed on an early version (September 2003) of the genome build [2] while the map-based finished quality sequence and its corresponding annotation of the rice genome became available in January 2005 [3]. Unlike the current genome build of *Arabidopsis *which is relatively stable, there have been significant changes across the various rice genome builds. The consequence is that there are several gaps on the tile path and further, a small but significant percentage of the probes, were lost, shuffled, map to multiple locations on the genome, or overlap existing probes. These factors are likely to affect the accurate detection of transcripts in rice and are likely to affect transcript coverage. The design of tiling arrays brings in issues that have implications on transcript coverage. Excluding repetitive DNA elements and other non-unique sequences is an important step when selecting sequences to be represented on a tiling array. As sequence tiles increase in size, the sequence fragmentation introduced by repetitive elements reduces the coverage of non-repetitive DNA [4,5]. Thus, tiling arrays are likely to miss transcripts that arise from regions of the chromosome that contain repetitive DNA and non-repetitive regions that are missed by the algorithm. In addition, transcript boundaries from processing tiling array data are currently assigned based on the start and end coordinates of probes represented on the array rather than the actual transcript boundaries.

The differences in transcript detection cannot be entirely attributed to the two technologies *per se*. The biological samples used in the four experiments (two tiling array and two MPSS sequencing) are processed in different laboratories and there is some overlap in the type of libraries used for RNA preparation. However, the precise effect of the differences due to the nature of biological sample variation on differential expression is difficult to delineate for the following reasons. For the *Arabidopsis *tiling array experiment, mRNA was extracted from T87 cultured cell line; the MPSS tags were obtained by a different group from sequencing 17 libraries constructed using mRNA from diverse tissues, mutants and treatments and does not include the T87 cultured cell line. For the Rice tiling array experiment RNA preparation involved pooled mRNA (not individual libraries) extracted from seedling root, seedling shoot, panicle and suspension-cultured cells. MPSS transcripts for the Rice experiment were obtained from sequencing 22 poly-adenylated mRNA libraries (see references in Additional File 1). These include libraries from 12 different untreated tissues and six abiotic stress treatments. Thus, the lack of matching tiling array data for the corresponding libraries used in the MPSS experiment makes it difficult to address how well the two methods compare with respect to differential expression.

## Competing interests

The authors declare that they have no competing interests.

## Authors' contributions

RS formulated the project, RS processed and analyzed the data and wrote the paper; AA did some of the annotation comparison; AA, JR and MG provided valuable comments for the project. All authors read and approved the final manuscript.

## Supplementary Material

Additional file 1 **Materials and methods**. This file describes the processing of tiling array and MPSS datasets for Arabidopsis and Rice. The file can be opened using Microsoft Word. Click here for file

Additional file 2 **Comparison of gene structures identified from MPSS and tiling microarray data for *Arabidopsis *and rice**. This file describes the methods used to identify the number of transcribed protein-coding gene loci from tiling array and MPSS datasets for Arabidopsis and Rice. The file can be opened using Microsoft Word. Click here for file

Additional file 3 **Distribution of intensities for unique MPSS tags for Arabidopsis and rice**. Panel A shows the distribution of intensities for 118,801 unique MPSS tags in *Arabidopsis *and Panel B shows the distribution of intensities for 68,413 unique MPSS tags in rice. The file can be opened using Adobe Acrobat Reader. Click here for file

Additional file 4 **Correlation of transcript abundance from MPSS data and intensity from tiling microarray data for Rice**. This file provides regression plots of log2 transformed abundance measure for MPSS tags against mean intensity percentile of MPSS tags calculated from tiling array data for the 22 libraries in rice. The name of the library and the correlation coefficient are given in the top right corner for each plot. The file can be opened using Microsoft Word. Click here for file

Additional file 5 **Correlation of transcript abundance from MPSS data and intensity from tiling microarray data for Arabidopsis**. This file provides regression plots of log_2 _transformed abundance measure for MPSS tags against mean intensity percentile of MPSS tags calculated from tiling array data for the 17 libraries in Arabidopsis. The name of the library and the correlation coefficient are given in the top right corner for each plot. The file can be opened using Microsoft Word. Click here for file
