# Supplementary material for: An approach to comparing tiling array and high throughput sequencing technologies for genomic transcript mapping
Source: BMC Res Notes. 2009 Jul 24;2:150. doi: 10.1186/1756-0500-2-150 (PMC2764720; doi:10.1186/1756-0500-2-150)
Supplement: Additional file 1 — Materials and methods. This file describes the processing of tiling array and MPSS datasets for Arabidopsis and Rice. The file can be opened using Microsoft Word. [file 1756-0500-2-150-S1.doc]

# Materials and Methods

## Tiling microarray and MPSS datasets

We obtained tiling microarray datasets for *Arabidopsis* *thaliana* (Arabidopsis) and *Oryza sativa* spp japonica cv. Nipponbare (rice) from National Center for Biotechnology Information’s (NCBI) Gene Expression Omnibus (GEO) database [1, 2], the series files are named GSE2247 and GSE6996, respectively. There are two datasets for Arabidopsis, the first Arabidopsis tiling array results from Yamada et al. in 2003 [3] and the second set of arrays that were described in 2005 by Stolc et al. [4]. We decided to use the latter as it is similar in design to the rice tiling array.

The details of the Maskless Array Synthesizer (MAS) technology microarray design and hybridization for Arabidopsis genome tiling array experiment is described here [4]. Briefly, 13 high-density arrays were constructed to represent the five nuclear chromosomes and the genomes of mitochondria and chloroplast. A total of ~5M 36-mer oligo-nucleotide probes, positioned every 46nt on average, were selected uniformly from the two strands of the non-repetitive region of chromosomal sequences (January 2002 release). The probes were synthesized at a feature density of about 400,000 probes per array. The array was hybridized with fluorescence-labelled cDNA reverse-transcribed from poly-adenylated mRNA extracted from T87 cultured cell line. All the probes were remapped to the current release (The Arabidopsis Information Resource (TAIR) version 7) of Arabidopsis genome. The corresponding annotation file (TAIR7 gff3) was used for downstream analysis. Less than 0.1% of the total ~5 million probes were lost or mapped to multiple locations in the genome. The chromosome sequence files and the annotation file were downloaded from TAIR’s ftp site [5].

The array design for rice is similar to the one described above for Arabidopsis [6]. To summarize, 32 high-density arrays were constructed to represent a total of ~12.2 million probes chosen from the 12 chromosomes of the rice genome (TIGR Rice Genome Annotation Release 1, September 2003 [7]). The rice array was hybridized with pooled mRNA extracted from seedling root, seedling shoot, panicle and suspension-cultured cells. The chromosome sequences and the annotation file were obtained from the The Institute for Genome Resource’s (TIGR) ftp site [8]. We were able to map 95% of the 12.2 million total probes to the current version of the Rice genome (version 5); a small fraction of the probes mapped to more than five locations in the genome. These probes were not considered for the work described here. Although the Arabidopsis and rice tiling microarray datasets come from different laboratories, both the arrays were constructed using the NimbleGen™ MAS technology.

The MPSS (Massively Parallel Signature Sequencing) dataset for Arabidopsis and rice were obtained from Plant MPSS databases at Delaware Biotechnology Institute [9, 10]. The collection of rice MPSS dataset represents 249,990 distinct transcripts in the form of short 17bp tags obtained from sequencing 22 poly-adenylated mRNA libraries. These include libraries from 12 different untreated tissues and six abiotic stress treatments [11]. A set of 121,581 reliable tags from this collection were mapped to the current version of the rice genome. Of the 80,978 tags that mapped to the genome, 68,413 tags mapped to a unique location and the rest 12,565 tags mapped to two or more locations. 11,633 of the 12,565 tags mapped to two to ten locations. In total, we considered 100,274 tags or tag locations considering the ones that map to multiple locations (up to 10 times) for downstream analysis. 40,603 of the total reliable tags were lost as they could not be mapped to any location in the genome.

To analyse MPSS transcripts from Arabidopsis, we started with a set of 118,801 reliable tags obtained from the *Arabidopsis* MPSS database. These transcripts are derived from 17 libraries constructed using mRNA from diverse tissues, mutants and treatments [12]. As before for rice, these tags were mapped to the current version of the *A*.*thaliana* genome build (TAIR version 7). 107,070 of the 118,801 tags mapped to a unique genomic location. 8,956 of the remaining 9,128 tags mapped to between two and ten genomic locations and these were considered. In total, we considered 128,337 tags or tag locations for downstream analysis. A total of 2,603 tags were lost as they could not be mapped to any location in the genome.

## Scoring computationally predicted gene structures on the tiling microarray

We used probe intensities from tiling array experiments for two goals. Given the set of computationally predicted exons and gene structures, how many genes can we identify as transcribed? We used binomial expansion, in the form of *sign-test*, to detect transcription in annotated gene models[13]. To accomplish this, we first identified probes that lie within the exons of a given gene model. For each probe, we checked if its intensity was greater than the median of the array (slide) it came from. For each gene model, we determined whether or not the number of probes that are above the median is more than expected by chance alone. The probability, p, of obtaining h probes with intensities above median out of N probes is given by the equation


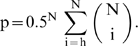


We chose a false-positive rate of 5%. Thus, gene models with a p-value less than 0.05 were scored as transcribed. We followed this procedure and scored computationally predicted gene structures in Arabidopsis and rice.

## Identifying transcriptionally active regions on the tiling microarray

Given the probe intensities and gene structure annotation, our second goal was to identify regions of transcription outside exons. These regions are also known as transcriptionally active regions (TARs) or transfrags [13, 14]. Since a probe or tile is too short to constitute a transcribed region, we employed the scoring scheme described in Cawley et al. [15] and Emanuelsson et al. [16] to identify TARs. In this procedure, qualified tiles that are close to each other along the genomic sequence are combined into features based on two parameters: (i) *maxgap* or maximum genomic distance below which two adjacent qualified tiles can be joined and (ii) *minrun*, the minimum length of a genomic region for it to be qualified as a feature. To identify TARs in Arabidopsis and rice, the corresponding tile path was scanned for regions in the genome that are covered by a *maxgap* of 20nt and a *minrun* of 174nt (at least four consecutive probes). Only probes whose intensities lie in the 85^th^ percentile or above were considered when scanning the tile path.

**Regression calculation of MPSS tag abundance *vs* tiling array intensities**

We used the mean intensity percentile for each of the MPSS tags as described above to see how well this measure of hybridization correlates with the abundance measurement of the respective tags obtained from the MPSS experiments. There are 17 libraries in *Arabidopsis* and 22 libraries in rice with an abundance measurement for all the reliable MPSS tags. We used normalized expression levels in "TPM" (transcripts per million) and not the raw tag abundance for the 17 base MPSS signatures. Hence, these expression measurements can be directly used for comparison without any further processing. These normalized expression levels were obtained from the MPSS database for Rice and *Arabidopsis* maintained at http://mpss.udel.edu by Blake Meyers’ group. A detailed description on how these normalized transcript abundance levels are arrived at is provided in the following reference [12].

In order to be conservative we considered only tags that map to a unique genomic location. We performed regression of the log_2_ transformed abundance values against the intensity percentile for unique MPSS tags in both rice (Additional File 3) and *Arabidopsis* (Additional File 4) and found that they are not correlated. This observation was consistent when we calculated the regression for individual libraries, pooled libraries and also for MPSS tags that overlap with tiling array TARs from the two species. The correlation coefficient for the pooled 17 libraries in *Arabidopsis* was 0.27 (range, 0.15-0.42) and 0.13 for MPSS tags that overlap with TARs. For rice, the correlation coefficient for the pooled 22 libraries was 0.25 (range, 0.13-0.26) and 0.13 for tags that overlap TARs. Regression of the log_2_ transformed abundance values against the intensity percentile for unique MPSS tags in both *Arabidopsis* and rice are not correlated. This suggests that the intensity value as a measure of hybridization as deduced from tiling array experiments cannot be confidently translated into measures of expression level for a given transcript. Further, the tag position bias observed for Classic and Signature MPSS methods compound this correlation.

# References
