# Supplementary material for: An approach to comparing tiling array and high throughput sequencing technologies for genomic transcript mapping
Source: BMC Res Notes. 2009 Jul 24;2:150. doi: 10.1186/1756-0500-2-150 (PMC2764720; doi:10.1186/1756-0500-2-150)
Supplement: Additional file 2 — Comparison of gene structures identified from MPSS and tiling microarray data for Arabidopsis and rice. This file describes the methods used to identify the number of transcribed protein-coding gene loci from tiling array and MPSS datasets for Arabidopsis and Rice. The file can be opened using Microsoft Word. [file 1756-0500-2-150-S2.doc]

## Comparison of gene structures identified from MPSS and tiling microarray

One of our first goals was to compare the extent of coverage of gene structure annotation between tiling microarray and MPSS platforms. The identification of gene models that are supported by MPSS tags is relatively straightforward but not obvious when using tiling array data. In order to identify transcribed genes on the tiling array we employed a scoring procedure that is statistically robust and consistent across the board. In the current version of annotation for *Arabidopsis* there are 31,712 gene models including alternatively spliced transcripts. We excluded 2,099 gene models that had less than 5 probes and/or had less than 45% of the total number of expected probes. This is because at least 5 probes are required to obtain a statistically significant p-value of being expressed and the latter set of genes may not be very well represented on the array. Since the resolution of the tiling array does not permit discriminating alternatively transcribed exons and gene structures, we considered the locus to be transcribed if at least one of the gene models has a p-value less than 0.05. This procedure resulted in 25,032 gene models that we evaluated for their transcription status, from which we identified 17,082 gene models or about 68% of all *Arabidopsis* genes as transcribed.

Now, with a reliable set of MPSS tags, we could investigate how many gene models were covered by this technology, After mapping the 128,337 reliable MPSS tags to the current version of the *Arabidopsis* genome build, we counted the number of tags that mapped to CDS, 5′ and 3′ UTR regions. We identified 21,053 genes (78.5%) that had at least one MPSS tag from the 26,819 gene structures that represent unique gene loci. On average, there are about 2.6 MPSS tags per gene structure.

Figure 1A shows the number of genes identified by the two methods and the overlap between them for *Arabidopsis*. There are 15,402 genes identified by both the methods. This overlap is 90.2% and 73.2% of all genes identified by the tiling microarray and MPSS platforms, respectively.

There are a total of 52,026 non-transposable-element related gene models in the rice genome. Out of this we excluded 5,850 gene models as they had less than 5 probes and/or had less than 45% of the total number of expected probes. As explained before for *Arabidopsis*, we considered the locus to be transcribed if at least one of the gene models has a p-value less than 0.05 [2]. Out of the 36,332 gene models that were evaluated for their transcription status we identified 21,219 gene models or 58.4% of all rice genes as transcribed.

We mapped the 100,274 reliable rice MPSS tags to the current version of the genome build and counted the number of tags that mapped to CDS, 5′-UTR and 3′-UTR regions. We identified 23,160 genes that had at least one MPSS tag from the 41,754 gene structures that represent unique gene loci. On average, there are about 2.4 MPSS tags per gene structure.

Figure 1B shows the number of genes identified by the two methods and the overlap between them for rice. There are 15,674 genes identified by both the methods. This overlap is 73.9% and 67.7% of all genes identified by tiling microarray and MPSS platforms, respectively. Thus, we observed a good overlap in the number of genes identified as transcribed by both the platforms. The overlap is higher in *Arabidopsis* than rice which is likely to be consistent irrespective of the scoring procedure applied. This is because the current state of annotation of the *Arabidopsis* genome is far more stable than the rice genome.

**15,402**

**17,082**

**21,053**

**MPSS**

**Tiling**

**Array**

***Arabidopsis***

**A**

**15,674**

**21,219**

**23,160**

**MPSS**

**Tiling**

**Array**

**Rice**

**B**

Figure 1

## Additional File 2, Figure 1 - Comparison of transcribed genes identified from tiling microarray and MPSS technologies for *Arabidopsis* and rice

The Venn diagrams in Figure 1A and 1B show the number of gene loci identified as transcribed from the two platforms and the overlap in the number of gene loci between the two methods for *Arabidopsis* and rice, respectively.
