# Supplementary material for: An approach to comparing tiling array and high throughput sequencing technologies for genomic transcript mapping
Source: BMC Res Notes. 2009 Jul 24;2:150. doi: 10.1186/1756-0500-2-150 (PMC2764720; doi:10.1186/1756-0500-2-150)
Supplement: Additional file 3 — Distribution of intensities for unique MPSS tags for Arabidopsis and rice. Panel A shows the distribution of intensities for 118,801 unique MPSS tags in Arabidopsis and Panel B shows the distribution of intensities for 68,413 unique MPSS tags in rice. The file can be opened using Adobe Acrobat Reader. [file 1756-0500-2-150-S3.pdf]

A

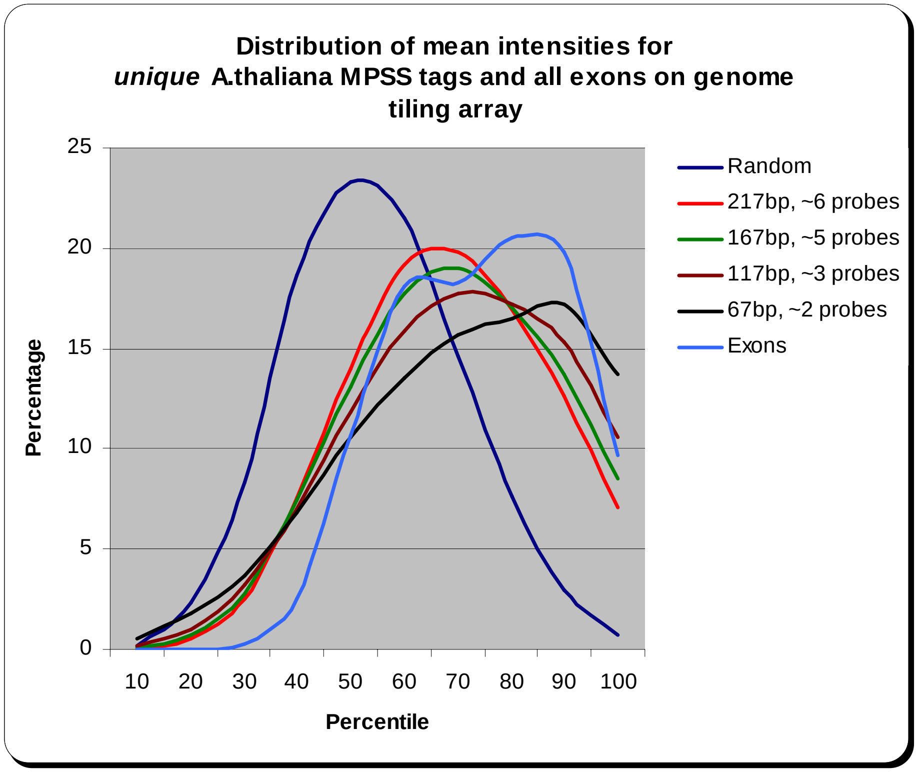

Distribution of mean intensities for *unique* A.thaliana MPSS tags and all exons on genome tiling array
Percentage
Percentile
Random
217bp, ~6 probes
167bp, ~5 probes
117bp, ~3 probes
67bp, ~2 probes
Exons

B

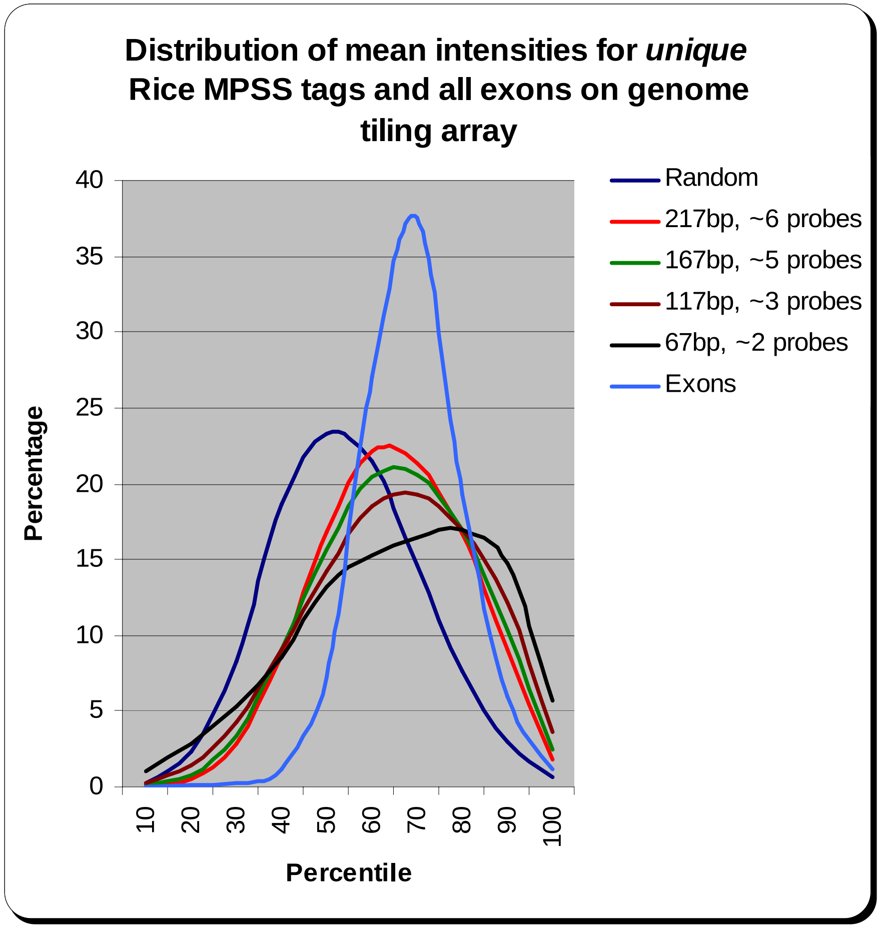

Distribution of mean intensities for *unique* Rice MPSS tags and all exons on genome tiling array
Percentage
Percentile
Random
217bp, ~6 probes
167bp, ~5 probes
117bp, ~3 probes
67bp, ~2 probes
Exons
