# Supplementary material for: An approach to comparing tiling array and high throughput sequencing technologies for genomic transcript mapping
Source: BMC Res Notes. 2009 Jul 24;2:150. doi: 10.1186/1756-0500-2-150 (PMC2764720; doi:10.1186/1756-0500-2-150)
Supplement: Additional file 5 — Correlation of transcript abundance from MPSS data and intensity from tiling microarray data for Arabidopsis. This file provides regression plots of log2 transformed abundance measure for MPSS tags against mean intensity percentile of MPSS tags calculated from tiling array data for the 17 libraries in Arabidopsis. The name of the library and the correlation coefficient are given in the top right corner for each plot. The file can be opened using Microsoft Word. [file 1756-0500-2-150-S5.doc]

## Additional file 5

## Regression plots of log_2_ transformed abundance for MPSS tags against mean intensity percentile of MPSS tags for the 17 libraries i*n* *Arabidopsis*. The name of the library and the correlation coefficient are given in the top right corner for each plot.

| A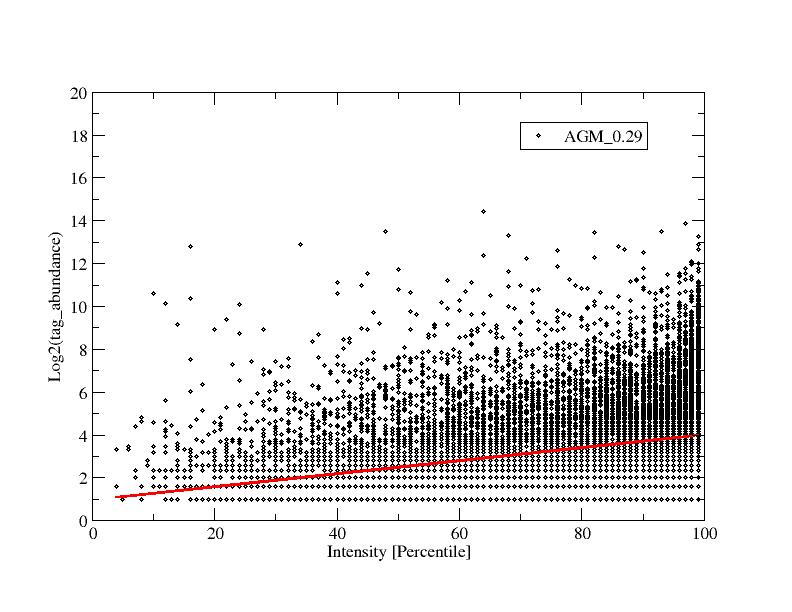 | B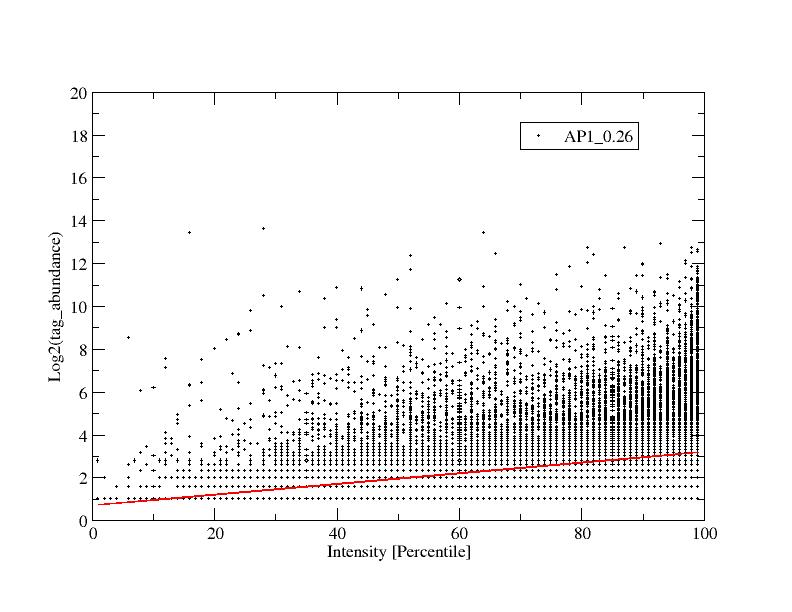 | |
| --- | --- | --- |
| C  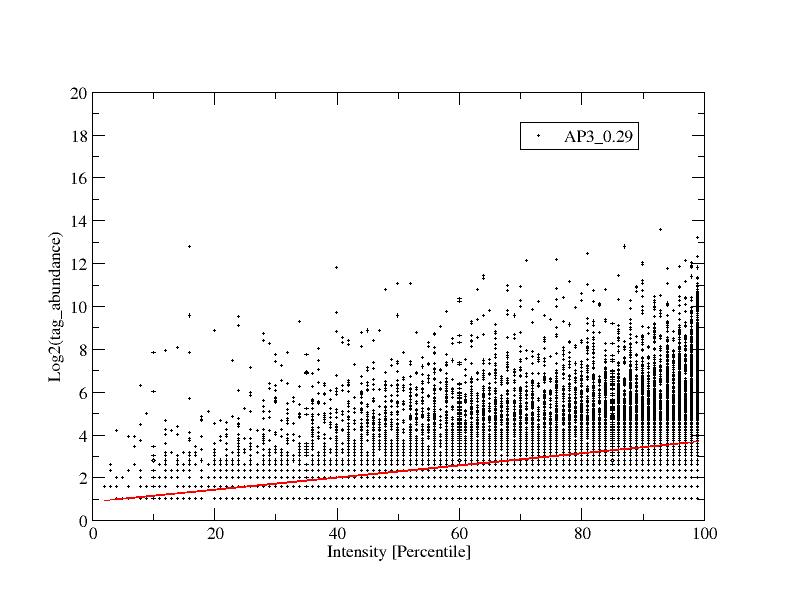 | D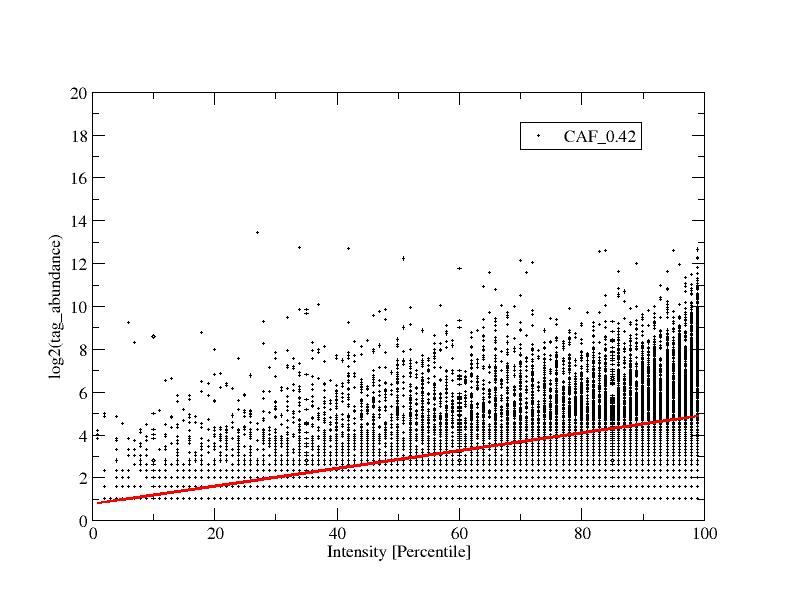 | |
| E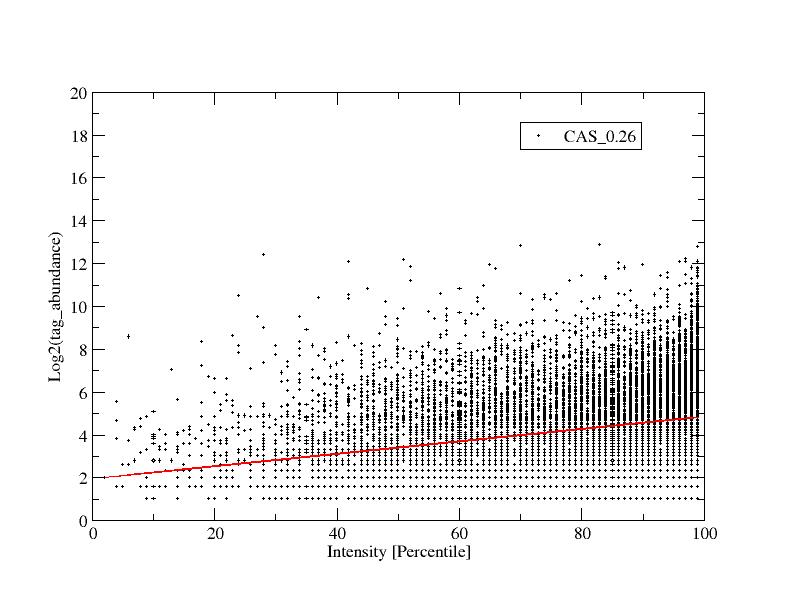 | F  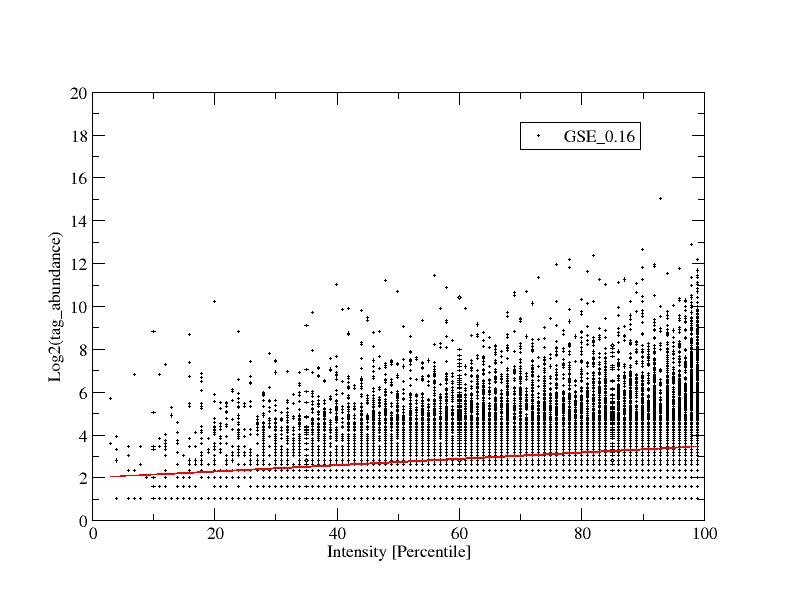 | |
| G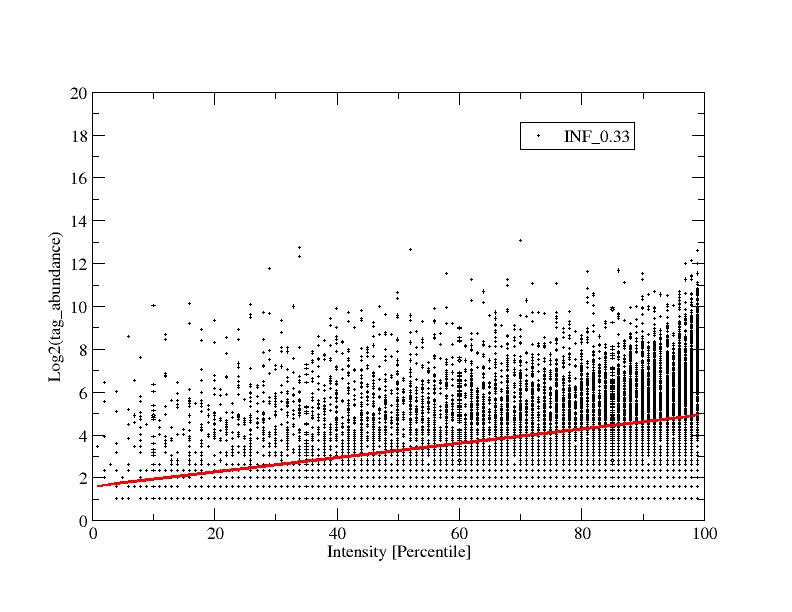 | H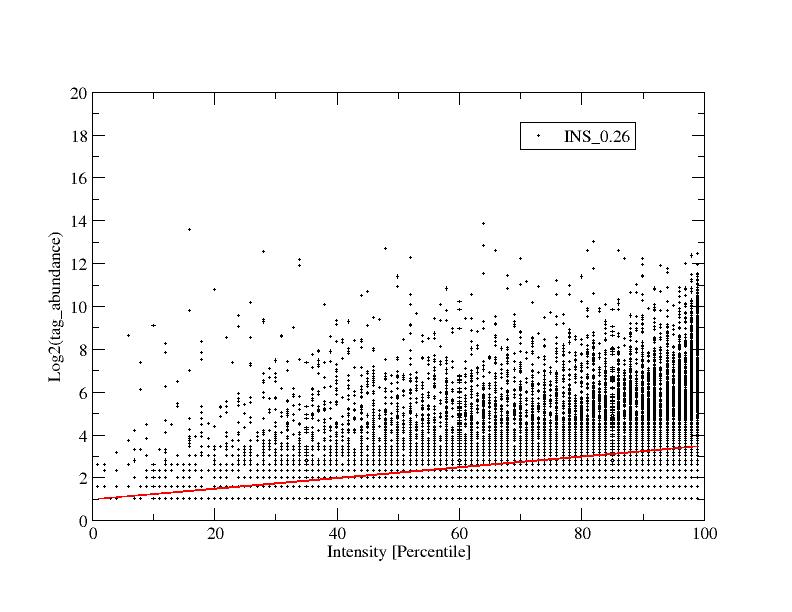 | |
| I  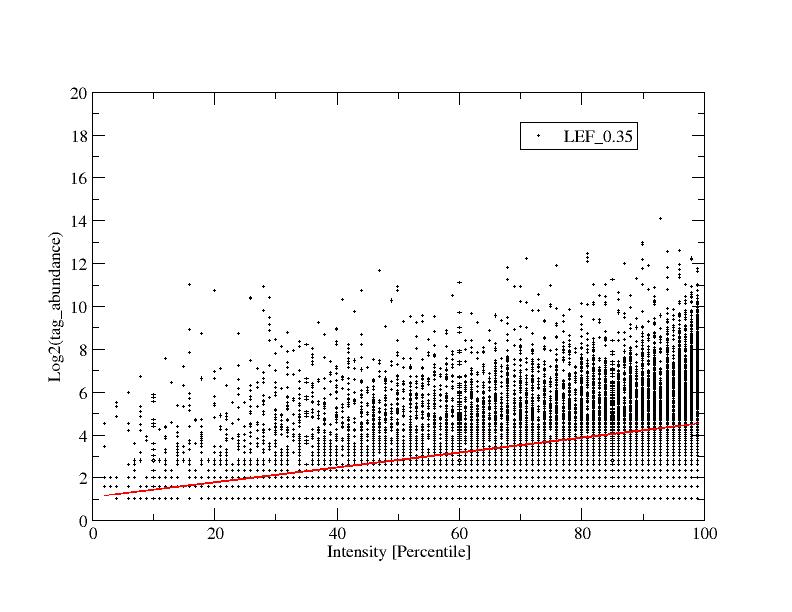 | J  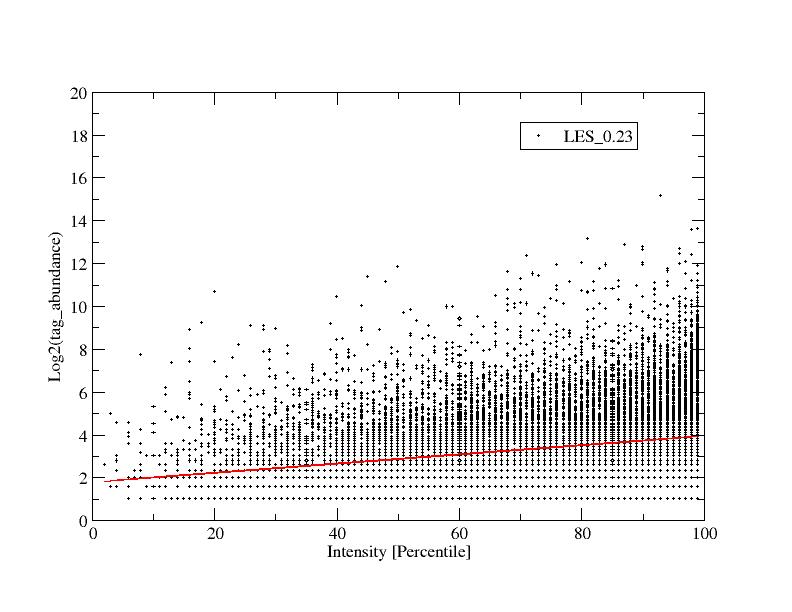 | |
| K  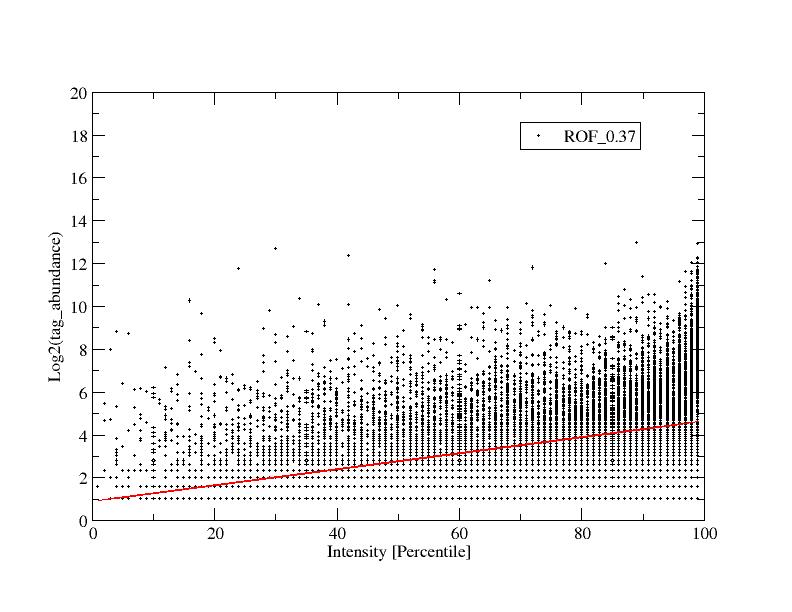 | L  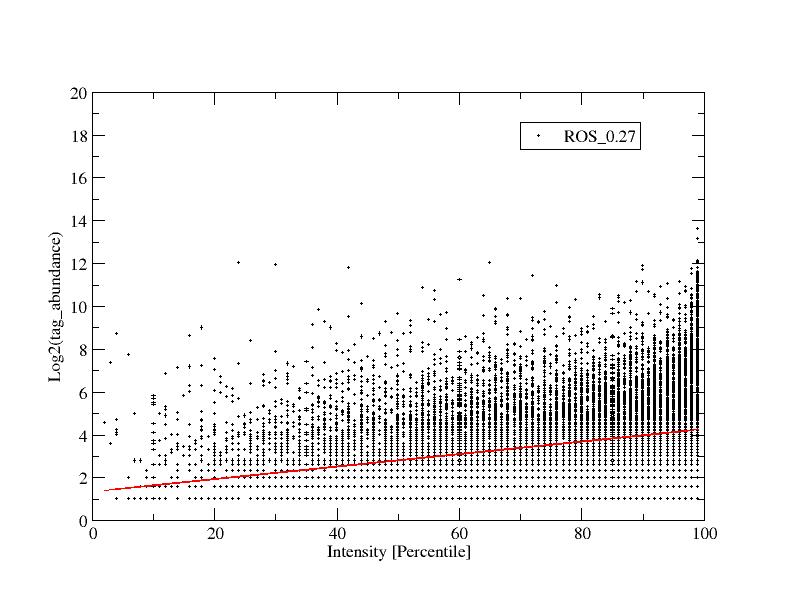 | |
| M  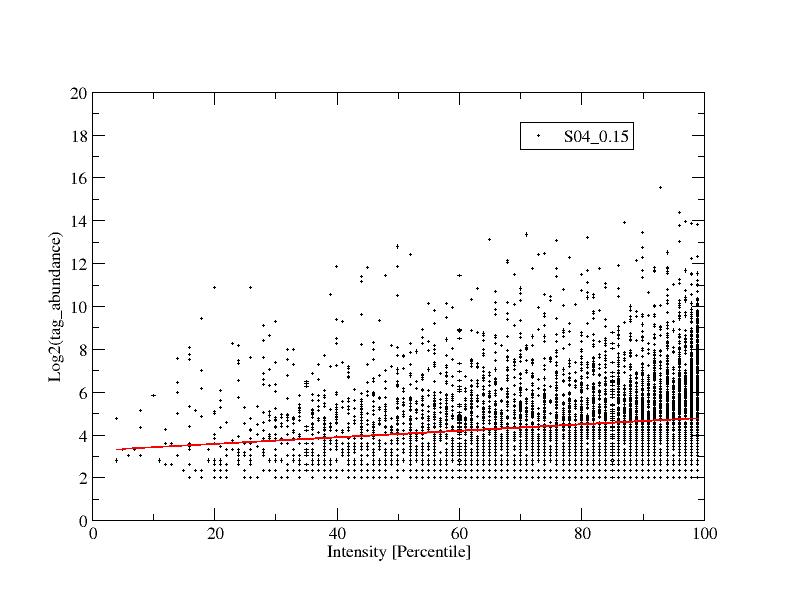 | N  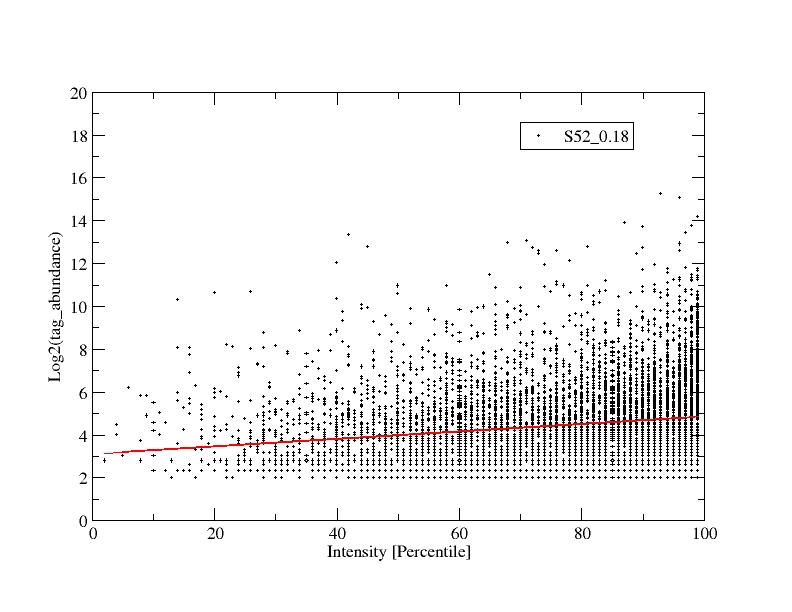 | |
| O  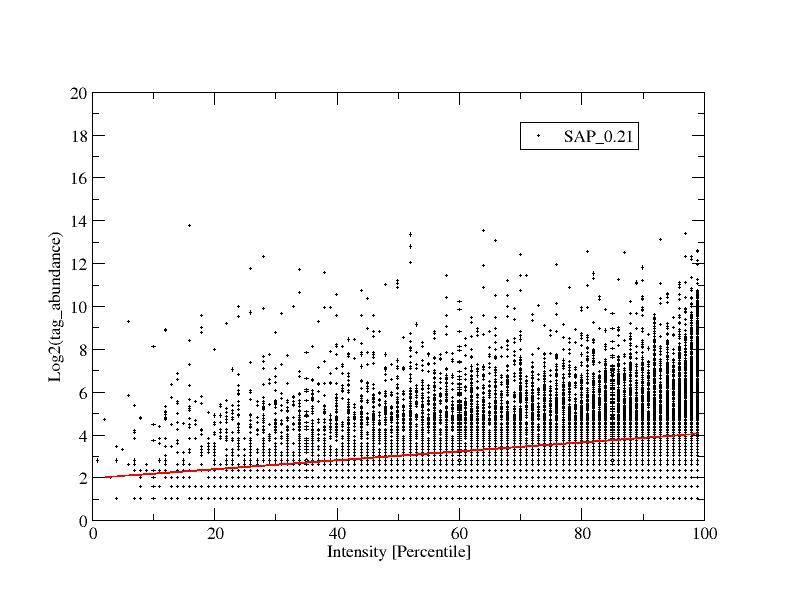 | P  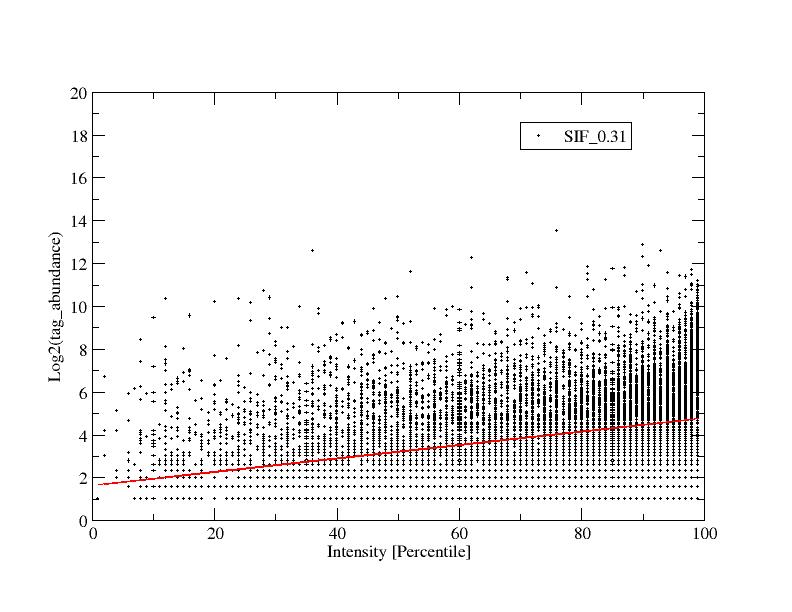 | |
| Q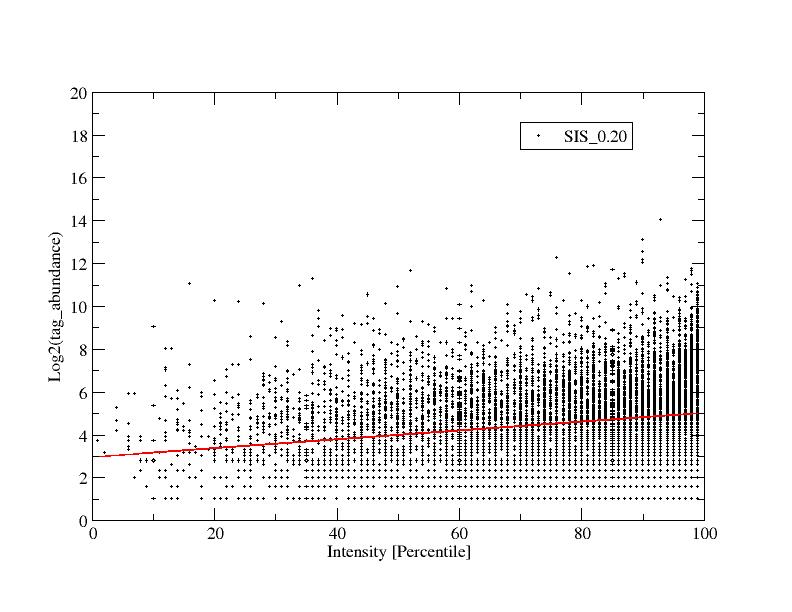 | R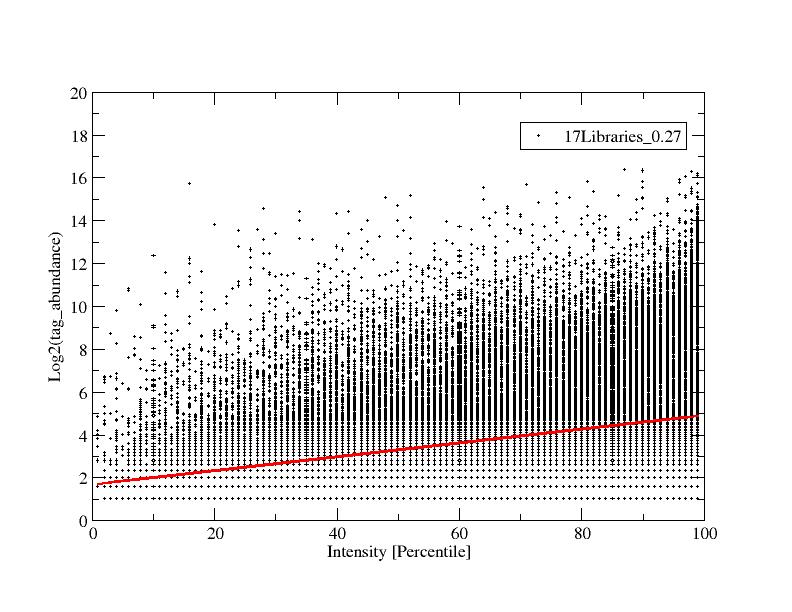 |  |
| S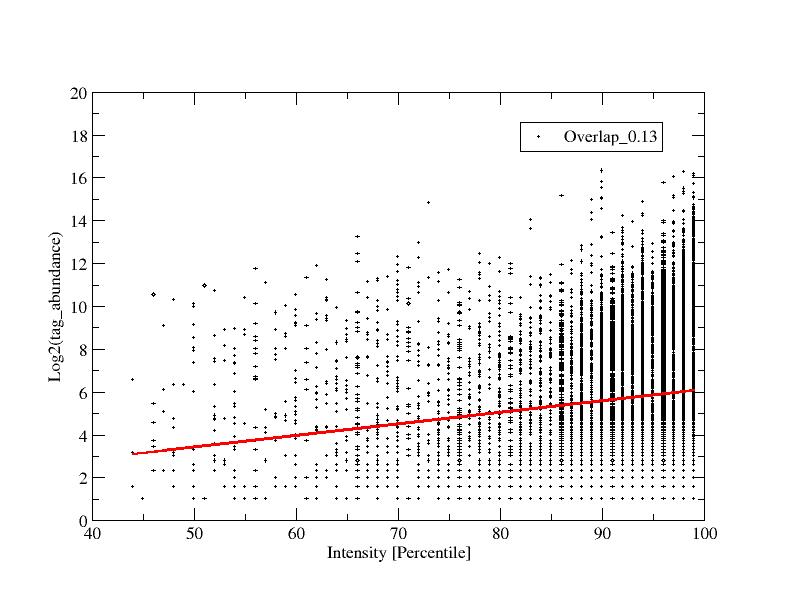 |  |  |
